# A Simple Fluorescent Cholesterol Labeling Method to Cryoprotect and Detect Plasma Lipoprotein-X

**DOI:** 10.3390/biology11081248

**Published:** 2022-08-22

**Authors:** Edward B. Neufeld, Lita A. Freeman, Vinay Durbhakula, Maureen L. Sampson, Robert D. Shamburek, Sotirios K. Karathanasis, Alan T. Remaley

**Affiliations:** Lipoprotein Metabolism Laboratory, National Heart, Lung, and Blood Institute, National Institutes of Health, Bethesda, MD 20892-1765, USA

**Keywords:** lipoprotein-X, familial LCAT deficiency, cholestasis, BODIPY-cholesterol, trehalose

## Abstract

**Simple Summary:**

Lipoprotein-X is an abnormal toxic particle in blood that is highly enriched in cholesterol. Lipoprotein-X forms in patients lacking an enzyme in blood called lecithin-cholesterol-acyl-transferase. With time, lipoprotein-X causes kidney disease in these patients, resulting in death at 40–50 years of age. Lipoprotein-X also forms, at very high levels, in the blood of patients with several different types of liver disease. Such high levels of lipoprotein-X cause additional painful and debilitating problems in these patients that can also be fatal. Currently, difficult and time-consuming tests only available in research laboratories can identify lipoprotein-X in blood. Unfortunately, lipoprotein-X in patient blood samples is unstable outside the body, and so with time becomes undetectable, even more so if it is frozen for evaluation at a later time. We have developed a simple method to label blood-derived lipoprotein-X so that it can be easily detected, and this method also stabilizes lipoprotein-X particles when frozen, enabling its detection after thawing. This methodology can easily be developed into a simple clinical test to identify both types of diseases where lipoprotein-X particles form in the blood and can be used to monitor how well treatments are able to reduce toxic lipoprotein-X in people with these diseases.

**Abstract:**

Lipoprotein-X (LpX) are abnormal nephrotoxic lipoprotein particles enriched in free cholesterol and phospholipids. LpX with distinctive lipid compositions are formed in patients afflicted with either familial LCAT deficiency (FLD) or biliary cholestasis. LpX is difficult to detect by standard lipid stains due to the absence of a neutral lipid core and because it is unstable upon storage, particularly when frozen. We have recently reported that free cholesterol-specific filipin staining after agarose gel electrophoresis sensitively detects LpX in fresh human plasma. Herein, we describe an even more simplified qualitative method to detect LpX in both fresh and frozen–thawed human FLD or cholestatic plasma. Fluorescent cholesterol complexed to fatty-acid-free BSA was used to label LpX and was added together with trehalose in order to cryopreserve plasma LpX. The fluorescent cholesterol bound to LpX was observed with high sensitivity after separation from other lipoproteins by agarose gel electrophoresis. This methodology can be readily developed into a simple assay for the clinical diagnosis of FLD and biliary liver disease and to monitor the efficacy of treatments intended to reduce plasma LpX in these disease states.

## 1. Introduction

Lipoprotein-X (LpX) is an abnormal toxic lipoprotein that markedly differs from normal lipoproteins in its structure, composition, and function. Instead of the micelle-like structure of normal lipoproteins, LpX is in the form of a vesicle, with an aqueous core surrounded by phospholipids and free cholesterol in either a bilayer-like or multilamellar configuration [1]. Its main protein component is albumin, which is trapped in the aqueous core, and it only contains a relatively small amount of exchangeable apolipoproteins on its surface [1]. It is present in plasma of familial lecithin-cholesterol-acyl-transferase (LCAT) deficiency (FLD) and cholestasis patients. LpX in familial LCAT deficiency (FLD) patients is nephrotoxic [2] and is associated with cloudy corneas, anemia, and the development of proteinuria, which can progress to nephrotic syndrome and end-stage renal disease in the fourth or fifth decade of life [3]. LpX has been shown to have a high predictive value for the diagnosis of cholestasis in liver cirrhosis [4]. In cholestatic patients, high concentrations of Lp-X can produce plasma hyperviscosity syndrome due to markedly elevated levels of plasma cholesterol (>1000 mg/dL) [5,6,7]. Complications associated with plasma LpX-mediated hyperviscosity include pulmonary embolism and/or pulmonary cholesterolomas [5,6]. Lp-X uptake by macrophages in the skin of cholestatic patients can also lead to the formation of planar xanthomata [4,8]. LpX has also been reported in patients who receive lipid-rich parenteral nutrition [9] and to cause severe disease in patients with cholestatic graft versus host disease of the liver after an allogeneic bone marrow transplant. Severe hypercholesterolemia from LpX is also an often-unrecognized long-term complication of allogeneic hematopoietic stem cell transplantation [10,11].

Due to its peculiar biophysical characteristics, it has been difficult to develop a suitable assay for the routine clinical detection of plasma LpX. Size exclusion chromatography, for example, cannot distinguish LPX from VLDL. Moreover, because LpX, unlike normal plasma lipoproteins, lacks a hydrophobic core containing triglycerides and cholesterol esters, it cannot be easily detected on agarose gels using conventional methods that stain the hydrophobic lipids, i.e., Sudan Black [1], which is the only method currently used by clinical laboratories. Recently, we described a sensitive method to detect LpX in fresh plasma by free-cholesterol-specific fluorescent filipin staining of agarose gels [12]. The lability of LpX during storage and the long duration of staining and expense of filipin are limitations of this method, which is also better suited as a research assay, because of the far UV wavelength needed to excite filipin [12]. Herein, we investigated two different fluorescent probes, namely, BODIPY-cholesterol and lissaminerhodamine phosphatidylethanolamine, in conjunction with trehalose, a cryopreservation agent [13,14], for the detection of LpX in either fresh or frozen plasma. Labeling plasma with BODIPY-cholesterol in the presence of trehalose was found to be preferable to cryoprotect LpX and is more amenable than previous staining methods for translation into a routine diagnostic assay.

## 2. Materials and Methods

### 2.1. Preparation of Synthetic Lp-X

Synthetic LpX was prepared as described in Ossoli et al., with minor modifications. Briefly, multilamellar LpX particles containing 24 mol % cholesterol were formed by combining 24.4 mg L-α-lecithin (Avanti Polar Lipids, Inc., Alabaster, AL, USA) with 4.25 mg cholesterol (Sigma Chemicals, St. Louis, MO, USA) from their respective stock solutions in chloroform and then drying the lipid mixtures under nitrogen. Two milliliters of normal saline was added to the dried lipids, and the mixture was vortexed and sonicated as described in the work of Ossoli et al. [2] to generate multilamellar particles enriched in cholesterol and phospholipid in the typical size range of LpX.

### 2.2. Lipoprotein Labeling with Fluorescent Lipids/Trehalose

Fluorescent lipid labeling solution was made by injecting fluorescent BODIPY-cholesterol (29 μL) and/or 65.1 μL lissaminerhodamine phosphatidylethanolamine, dissolved in ethanol, into 3 mL of saline containing 8 mg/mL fatty acid free-BSA, while vortexing. Human control, cholestatic or FLD patient plasma was incubated with fluorescent lipid labeling solution (100 μL) and/or 25 mM trehalose (from 500 mM stock solution in saline) in a total volume of 150 μL (after addition of saline) at 1200 RPM for 1 h at 37 °C in an Eppendorf ThermoMixer C. For freeze-thaw studies, samples were frozen at −70 °C overnight.

### 2.3. Agarose Gel Electrophoresis

Imaging of fluorescent-lipid-labeled lipoproteins [15] and filipin staining and imaging of lipoprotein cholesterol [12] on agarose gels was performed as previously described. Filipin-stained gels were photographed using the ethidium bromide filter, similar to photographing an ethidium-bromide-stained DNA gel using an AlphaImager’s UV light transilluminator (ProteinSimple, San Jose, CA, USA). BODIPY-cholesterol and lissaminerhodamine phosphatidylethanolamine fluorescence were detected using excitation/emission wavelengths of 488/520 nm and 532/560 nm, respectively.

### 2.4. Plasma Samples

EDTA-plasma collected from healthy (pooled) as well as FLD and cholestatic patients were stored on ice at 4 °C prior to labeling with fluorescent lipids and electrophoresis. All plasma samples used in this study were de-identified. The de-identified plasma samples were obtained from a study that was approved by the National Heart, Lung, and Blood Institute, Institute Review Board (protocol 03-H-0280) and is compliant with the Declaration of Helsinki principles. All subjects provided informed consent prior to participation in the study.

## 3. Results

### 3.1. BODIPY-Cholesterol Complexed to Fatty-Acid-Free BSA Labeled Synthetic and Human Cholestatic Plasma LpX

**Figure 1:**
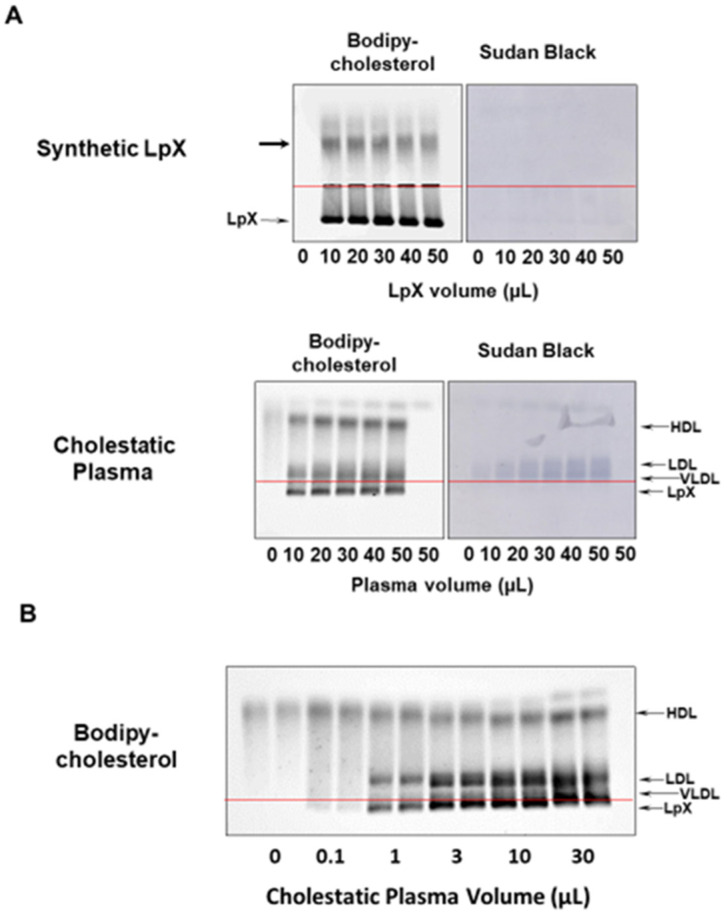
BODIPY-cholesterol complexed to fatty-acid-free BSA labeled synthetic and human cholestatic plasma LpX. Agarose gel electrophoresis. (**A**) Upper panel: Synthetic LpX was labeled following incubation with BODIPY-cholesterol complexed to fatty-acid-free BSA. The thick black arrow indicates excess BODIPY-cholesterol complexed to fatty-acid-free BSA that did not incorporate into the synthetic LpX. After imaging cholesterol fluorescence, gels were stained with Sudan Black (SB) for neutral lipids (cholesterol ester and triglycerides). Note that SB did not stain synthetic LpX, which lacks neutral lipids. Lower panel: Plasma from a cholestatic patient labeled with BODIPY-cholesterol complexed to fatty-acid-free BSA. Note that the cholestatic LpX, like synthetic LpX, did not stain with SB. (**B**) Cholestatic human plasma at the indicated volumes were incubated with BODIPY-cholesterol complexed with fatty-acid-free BSA. Duplicate samples were applied to the gel. Note the apparent plasma-volume-dependent labeling of cholestatic plasma LpX with BODIPY-cholesterol in the 1–10 µL range. The red lines in Figure 1, Figure 2, Figure 3, Figure 4 and Figure 5 indicate the origin.

BODIPY-cholesterol, a fluorescent cholesterol analog, was chosen as a potential label for LpX because it has been shown to mimic the partitioning of cholesterol between lipoproteins [15]. BODIPY-cholesterol complexed to fatty-acid-free BSA, after mixing with plasma, labeled both synthetic and endogenous LpX. After labeling with BODIPY-cholesterol, synthetic LpX (Figure 1A; Supplementary Materials Figure S1A) as well as LpX in cholestatic plasma (Figure 1A,B; Supplementary Materials Figure S1A,B) was readily detected on agarose gels after electrophoresis. Like LpX stained with filipin after agarose gel separation, the BODIPY-cholesterol-labeled LpX migrated toward the cathode, making it relatively easy to distinguish from other lipoproteins that also stained with the BODIPY-cholesterol probe. BODIPY-cholesterol labeling of cholestatic plasma was linear in the 1–10 µL plasma volume range. The more extensive migration of the synthetic LpX was likely due to the large size distribution of LpX particles in our preparations as well as compositional differences. BODIPY-cholesterol labeling of cholestatic plasma appeared to be linear in the 1–10 µL plasma volume range (Figure 1B; Supplementary Materials Figure S1A).

### 3.2. BODIPY-Cholesterol Labeling of Lipoproteins in Normal and Cholestatic Plasma Containing LpX Was Unaltered by Trehalose

**Figure 2:**
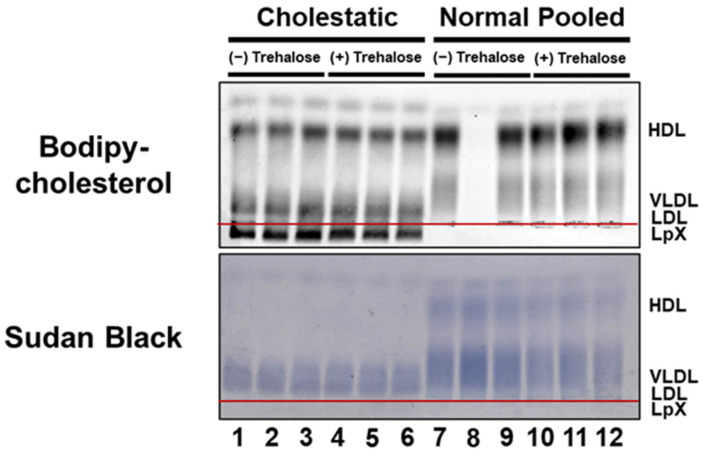
BODIPY-cholesterol labeling of lipoproteins in normal and cholestatic plasma containing LpX was unaltered by trehalose. Agarose gel electrophoresis. Cholestatic and normal pooled human plasma (25 μL) were incubated with BODIPY-cholesterol complexed to fatty-acid-free BSA in the absence ((−); Lanes #1–3; Lanes #7–9) or presence ((+); Lanes #4–6: Lanes #10–12) of the cryopreservation agent trehalose. Note that addition of trehalose did not alter either BODIPY-cholesterol labeling or Sudan Black (SB) staining, the electrophoretic mobility of LpX, or any other lipoproteins in normal or cholestatic plasma. Lane #8 contains unlabeled normal pooled human plasma for reference.

Another long-standing limitation for the routine measurement of plasma LpX is its instability, especially after freezing. We have long observed that FLD plasma LpX remains stable for only 2–3 days and becomes undetectable after approximately 10 days at 4 °C. Interestingly, we have observed in both our current and previous studies [12] that cholestatic plasma LpX tends to be more stable than FLD LpX at 4 °C, and that some cholestatic plasma LpX samples remain intact even after freezing. Still, this severely constrains routine clinical analyses, since patient plasma or serum samples are often frozen upon acquisition for future batch analysis. In order to address this problem, we examined whether the addition of trehalose, a cryopreservation agent, to plasma would stabilize plasma LpX from cholestatic plasma during freeze/thaw. First, we established that the addition of trehalose to either fresh normal or cholestatic plasma did not alter BODIPY-cholesterol labeling of either normal lipoproteins or LpX (Figure 2; Supplementary Materials Figure S2).

### 3.3. Combined Trehalose and BODIPY-Cholesterol Fatty-Acid-Free BSA Treatment Stabilized Cholestatic LpX during Freeze/Thawing

**Figure 3:**
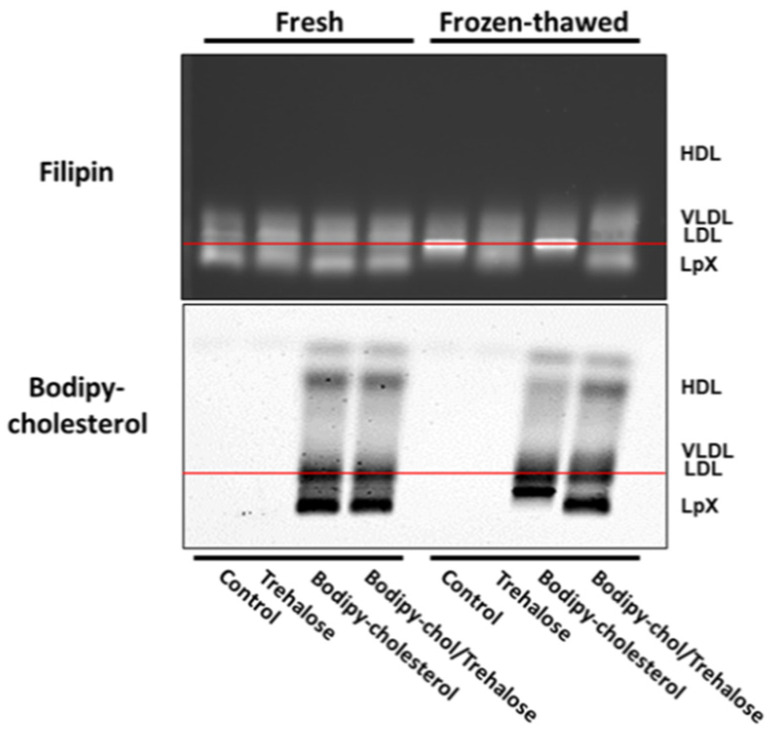
Combined trehalose and BODIPY-cholesterol fatty-acid-free BSA treatment stabilized cholestatic LpX during freeze/thawing. Agarose gel electrophoresis. Cholestatic human plasma (25 μL) was untreated (control), incubated with either trehalose alone (trehalose), or incubated with BODIPY-cholesterol complexed to fatty-acid-free BSA in the absence (BODIPY-cholesterol) or presence of trehalose (BODIPY-chol/Trehalose). Fresh and fresh/frozen samples were electrophoresed on agarose gels and then either stained with filipin and imaged or imaged for BODIPY fluorescence. Note that BODIPY-cholesterol alone (“BODIPY-cholesterol”, lower panel), trehalose alone (see Filipin, upper panel), or incubation with both trehalose and BODIPY-cholesterol (see “Filipin” and “BODIPY-cholesterol” panels) stabilized LpX in freeze–thawed cholestatic human plasma.

Next, we assessed whether addition of trehalose to plasma alone or labeling of LpX from cholestatic plasma with fluorescent cholesterol in the presence or absence of trehalose would stabilize LpX during freeze/thawing. Staining with BODIPY-cholesterol alone without trehalose resulted in the apparent aggregation of LpX (Figure 3; Supplementary Materials Figure S3). It remained trapped in the well of the gel, which often results in the sporadic loss of signal from diffusion into the gel buffer. In marked contrast, when the staining with BODIPY-cholesterol was conducted in the presence of trehalose, LpX was preserved after freeze–thawing and the freezing did not alter its electrophoretic mobility (Figure 3).

### 3.4. Combined Trehalose Treatment and Fatty-Acid-Free BSA-Mediated Labeling with BODIPY-Cholesterol, Lissaminerhodamine Phosphatidylethanolamine, or Both Stabilized Cholestatic LpX

**Figure 4:**
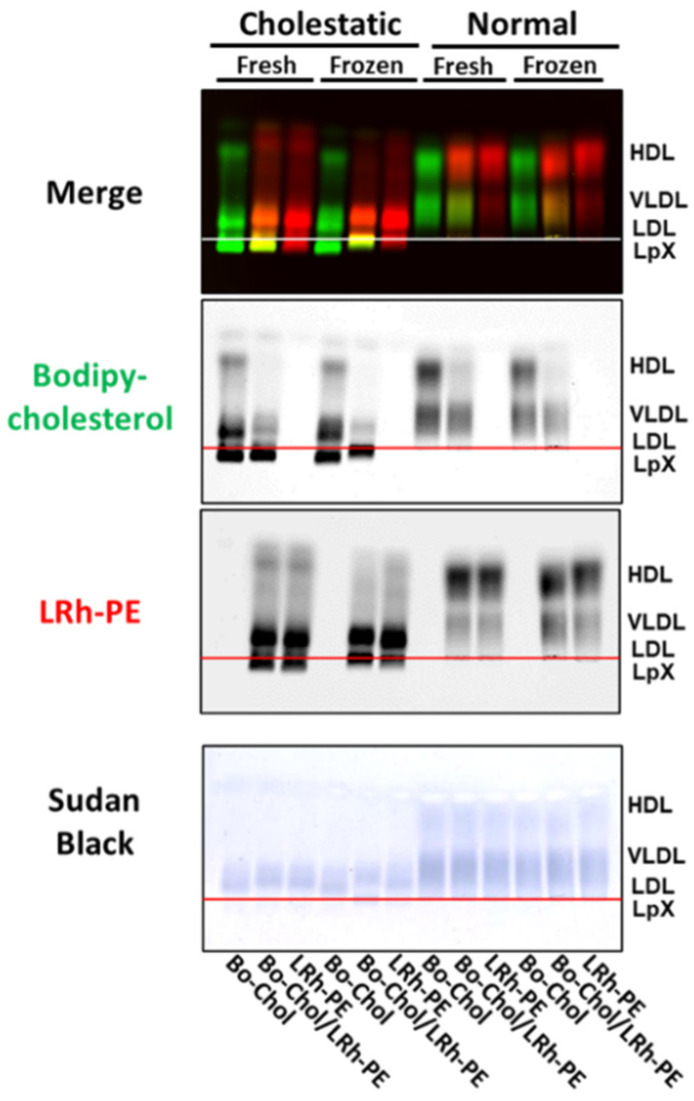
Combined trehalose treatment and fatty-acid-free BSA-mediated labeling with BODIPY-cholesterol, lissaminerhodamine phosphatidylethanolamine, or both stabilized cholestatic LpX. Agarose gel electrophoresis. Fresh or frozen/thawed cholestatic (25 μL) and normal human plasma (25 μL) were incubated with trehalose and either BODIPY-cholesterol (Bo-chol; green) and/or head-group labeled lissaminerhodamine phosphatidylethanolamine (LRh-PE; red) complexed to fatty-acid-free BSA. Overlapping BODIPY-cholesterol and lissaminerhodamine-labeled phosphatidylethanolamine appears orange-yellow in the Merge panel. After imaging fluorescence, gels were stained with Sudan Black (SB).

Because LpX contains phospholipids in addition to cholesterol, we also examined whether lissaminerhodamine phosphatidylethanolamine, a fluorescent phospholipid analog, could be used to detect LpX. We did this by comparing the labeling of normal and cholestatic plasma lipoproteins with BODIPY-cholesterol to labeling with lissaminerhodamine phosphatidylethanolamine alone or in combination with BODIPY-cholesterol in the presence of trehalose. As shown in Figure 4 (Supplementary Materials Figure S4), lissaminerhodamine phosphatidylethanolamine appeared to quench BODIPY-cholesterol labeling of LDL and HDL, but not LpX, on agarose gels. This observation suggests that the distribution of lissaminerhodamine phosphatidylethanolamine and BODIPY-cholesterol in LpX membranes differs from the distribution of these lipids on the surface monolayer of LDL and HDL. Intriguingly, lissaminerhodamine phosphatidylethanolamine labeling of LpX alone or in combination with BODIPY-cholesterol altered LpX migration on agarose gels in freeze–thawed, but not fresh, human cholestatic plasma. Taken together, these findings indicate that BODIPY-cholesterol BSA/trehalose is the preferred method for detecting LpX because it preserves the electrophoretic migration of cholestatic plasma LpX in both fresh and frozen plasma.

### 3.5. Combined Trehalose and BODIPY-Cholesterol Fatty-Acid-Free BSA Treatment Stabilized FLD LpX

**Figure 5:**
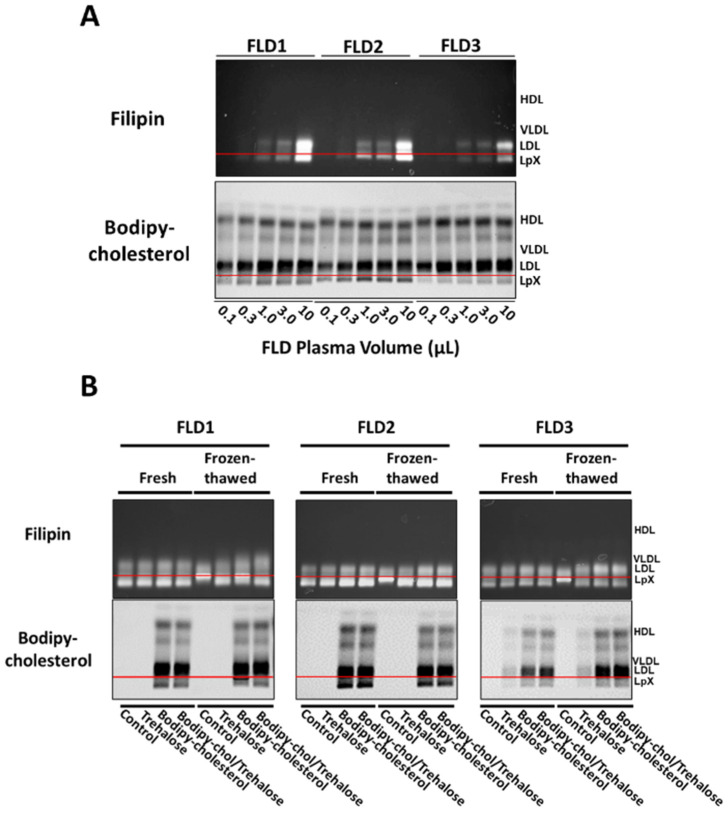
Combined trehalose and BODIPY-cholesterol fatty-acid-free BSA treatment stabilized FLD LpX. Agarose gel electrophoresis. (**A**) Plasma from three FLD patients was incubated with BODIPY-cholesterol complexed to fatty-acid-free BSA in the presence of trehalose. FLD plasma samples, using the volumes shown (0.1–10 µL), were electrophoresed on agarose gels and then imaged for BODIPY fluorescence or stained with filipin and imaged. Note that BODIPY-cholesterol labeling of LpX in all three FLD samples appeared to be more sensitive than filipin staining. BODIPY-cholesterol labeling was linear from 0.1 to 1 µL of FLD plasma. (**B**) FLD plasma (25 μL) from the same three patients were not treated (Control) or incubated with either trehalose alone (Trehalose) or with BODIPY-cholesterol complexed to fatty-acid-free BSA in the absence (BODIPY-cholesterol) or presence (BODIPY-chol/Trehalose) of trehalose. Fresh and fresh/frozen samples were electrophoresed on agarose gels and then stained with filipin or imaged for BODIPY fluorescence. Note that LpX was stabilized in freeze–thawed FLD human plasma by either BODIPY-cholesterol alone, trehalose alone (Filipin panel) or by co-incubation with trehalose and BODIPY-cholesterol (Filipin panel; BODIPY-cholesterol panel). Note the weak signal for BODIPY fluorescence in FLD3 samples treated with trehalose alone. This likely is due to low-level autofluorescence.

We then tested the ability of the BODIPY-cholesterol/BSA trehalose labeling method to detect LpX in FLD patient plasma (Figure 5; Supplementary Materials Figure S5). We first compared the sensitivity of BODIPY-cholesterol versus filipin labeling of FLD plasma LpX in the plasma from three different FLD patients. Both methods resulted in about the same relative differences in LpX levels in the plasma of the three different FLD patients. Importantly, the sensitivity of BODIPY-cholesterol detection of LpX appeared to be at least threefold higher than that of filipin insofar as BODIPY-cholesterol fluorescence intensity was much greater than that of filipin at the lowest levels of LpX tested (0.1–3.0 µL of FLD plasma).

Finally, we investigated whether incubation of FLD plasma with BODIPY-cholesterol alone or in combination with trehalose would stabilize FLD LpX during freeze/thaw (Figure 5B; Supplementary Materials Figure S5B). In order to assess the effect of trehalose itself on LpX stability, we also visualized non-BODIPY-cholesterol-labeled FLD LpX on filipin-stained agarose gels. After a single freeze/thaw, filipin staining revealed that trehalose alone stabilized FLD LpX (Figure 5B; Supplementary Materials Figure S5B). Moreover, after a single freeze/thaw, LpX in FLD plasma labeled with only BODIPY-cholesterol was also somewhat stabilized. Notably, LpX had similar electrophoretic mobility as LpX in FLD plasma treated with either trehalose alone or in combination with BODIPY-cholesterol. These findings differ from those in our studies above using cholestatic plasma (Figure 3; Supplementary Materials Figure S3), wherein BODIPY-cholesterol-labeled LpX, in the absence of trehalose, appeared to aggregate and remained trapped at the origin. This may reflect differences in LpX composition in FLD and cholestatic LpX, and/or perhaps the presence of other biliary components in cholestatic plasma that may alter the effect of BODIPY-cholesterol on LpX electrophoretic mobility. Taken together, these findings suggest that in order to ensure the optimal cryopreservation of LpX in plasma from either cholestatic or FLD plasma, it is best to include trehalose when labeling with BODIPY-cholesterol.

## 4. Discussion

To date, a simple routine clinical assay to detect and quantify lipoprotein-X (LpX) in human plasma has not been available. Sudan Black, which is a hydrophobic dye, has traditionally been used to image lipoproteins after separation by agarose gels. Because of its high hydrophobicity and lack of charged or polar groups, it enters into the hydrophobic core of lipoproteins. The degree of staining with Sudan Black is proportional to the cholesteryl ester and triglyceride content of lipoproteins. Because LpX is nearly devoid of cholesteryl ester and triglyceride, it does not stain very well with Sudan Black. We previously reported how filipin, a fluorescent bacterial antifungal polyene macrolide that binds to free cholesterol, can be used to detect LpX and is more sensitive than Sudan Black staining [12]. A limitation of filipin staining, besides its expense, is that the filipin staining solution can be stored for only a few days. In addition, the staining of the gel with filipin typically takes several hours and requires excitation with a laser that emits in the far UV range, which is not commonly available in routine clinical laboratories.

Another challenge is that LpX is labile, but the mechanism for this is uncertain. Upon storage, it has been shown that free cholesterol on lipoproteins and LpX can undergo esterification by LCAT, which may account for its loss with storage in plasma with LCAT activity. The absence of surface apolipoproteins may also make it prone to fusion and aggregation. It has been well documented that LpX is especially labile to freeze–thawing, which we have been able to prevent by the addition of trehalose. The altered migration of cholestatic or FLD plasma LpX treated with BODIPY-cholesterol alone indicates that BODIPY-cholesterol alone affords some cryoprotection to LpX (but not as much as trehalose alone, or in combination with trehalose). It has been proposed that the aqueous core forms ice crystals after freezing, causing aggregation or disruption of the LpX structure, which may account for why a cathodal migrating band from agarose gel electrophoresis from LpX typically disappears after freezing. Trehalose appears to interact with phospholipid polar head groups and stabilize lipid monolayers during freeze/thaw [16], apparently by interlocking several lipid molecules that simultaneously hydrogen bond to the same disaccharide molecule [17]. Cryoprotective agents such as trehalose have been proposed to modulate lipid phase transitions by decreasing the rate of membrane dehydration during freezing in platelets [14].

Both the cholesterol and phospholipid probes we tested efficiently labeled LpX, but we favor the use of BODIPY-cholesterol because it did not change the electrophoretic migration of LpX as we observed for lissaminerhodamine phosphatidylethanolamine. The different types of lipoproteins can be separated by agarose gel electrophoresis because of differences in their size and charge [18]. Because LpX is largely devoid of apolipoproteins, it is believed to be relatively neutral in charge and migrates toward the cathode, because of electroendosmosis [19]. Unlike BODIPY-cholesterol or cholesterol itself, lissaminerhodamine phosphatidylethanolamine has a net negative charge, which probably accounts for why staining LpX with this probe caused it to shift its migration more toward the anode.

In summary, we report on the development of a simple method to both label and cryopreserve LpX in plasma from either cholestatic or familial LCAT deficiency (FLD) patients. Patient plasma is incubated with trehalose and fluorescent BODIPY-cholesterol, after which it can be used fresh, or after freeze/thaw, to detect LpX-associated fluorescence via agarose gel electrophoresis. This methodology has the potential to be converted into a routine clinical assay to assess the severity and progression of FLD or cholestatic liver disease and to assess the efficacy of treatments to reduce plasma LpX in FLD and cholestatic liver disease. The two key reagents that we use to label LpX, namely, BODIPY-cholesterol and trehalose, are relatively inexpensive. Our labeling reagent is simply added to plasma and the sample can either be analyzed immediately or later on a frozen sample.

A limitation of our method is that it is currently only a semi-quantitative method, but for most clinical purposes, only the presence or absence of LpX is needed for managing patients. Another limitation is that for routine diagnostic testing this new method would have to be considered a “laboratory-developed test”, because it is not FDA approved, which would require clinical laboratories to do their own in-house validation (https://www.fda.gov/medical-devices/in-vitro-diagnostics/laboratory-developed-tests (accessed on 8 August 2022). Other gel systems for separating lipoproteins composed of a similar gel matrix and buffer will likely also work with our method, but this will need to be established in the future. Although our method will likely be unaffected by common laboratory interfering conditions/substances from hemolysis or cholestasis, very hyperlipidemic samples with elevated chylomicrons, which become trapped in the origin of the gel, could potentially interfere and may need to be first removed before analysis. Before our method could be implemented for routine diagnostic purposes, long-term stability of treated plasma samples at −70 °C will also need to be assessed. Despite its current limitations, our new method is relatively easy to perform and can be easily used at this time for research testing, and with some additional work could be developed into a routine diagnostic assay.

## 5. Conclusions

To date, toxic abnormal LpX found in FLD and cholestatic patient plasma can only be reliably measured using a difficult and time-consuming assay based on staining free cholesterol present in LpX [12]. The filipin assay is valuable research tool but is not amenable to translation to a routine clinical assay. Moreover, plasma LpX is unstable and so the detectable amount of plasma LpX decreases with time and particularly with freeze/thawing. We have circumvented these limitations by developing a simple method to label, detect, and cryoprotect LpX in both FLD and cholestatic plasma that can readily be developed into a routine clinical assay.

## Data Availability

Not applicable.

## Supplementary Materials

The following supporting information can be downloaded at: https://www.mdpi.com/article/10.3390/biology11081248/s1, Original Scans of Gels in Figures S1–S5.
