# Supplementary material for: A Simple Fluorescent Cholesterol Labeling Method to Cryoprotect and Detect Plasma Lipoprotein-X
Source: Biology (Basel). 2022 Aug 22;11(8):1248. doi: 10.3390/biology11081248 (PMC9405255; doi:10.3390/biology11081248)
Supplement: Supplementary file 1 [file biology-11-01248-s001.zip › biology-1838030-supplementary.pdf]

SUPPLEMENTARY MATERIALS

ORIGINAL SCANS OF GELS IN FIGURES S1-S5

Figure S1A

BODIPY-Cholesterol Fluorescence

Sudan Black Staining

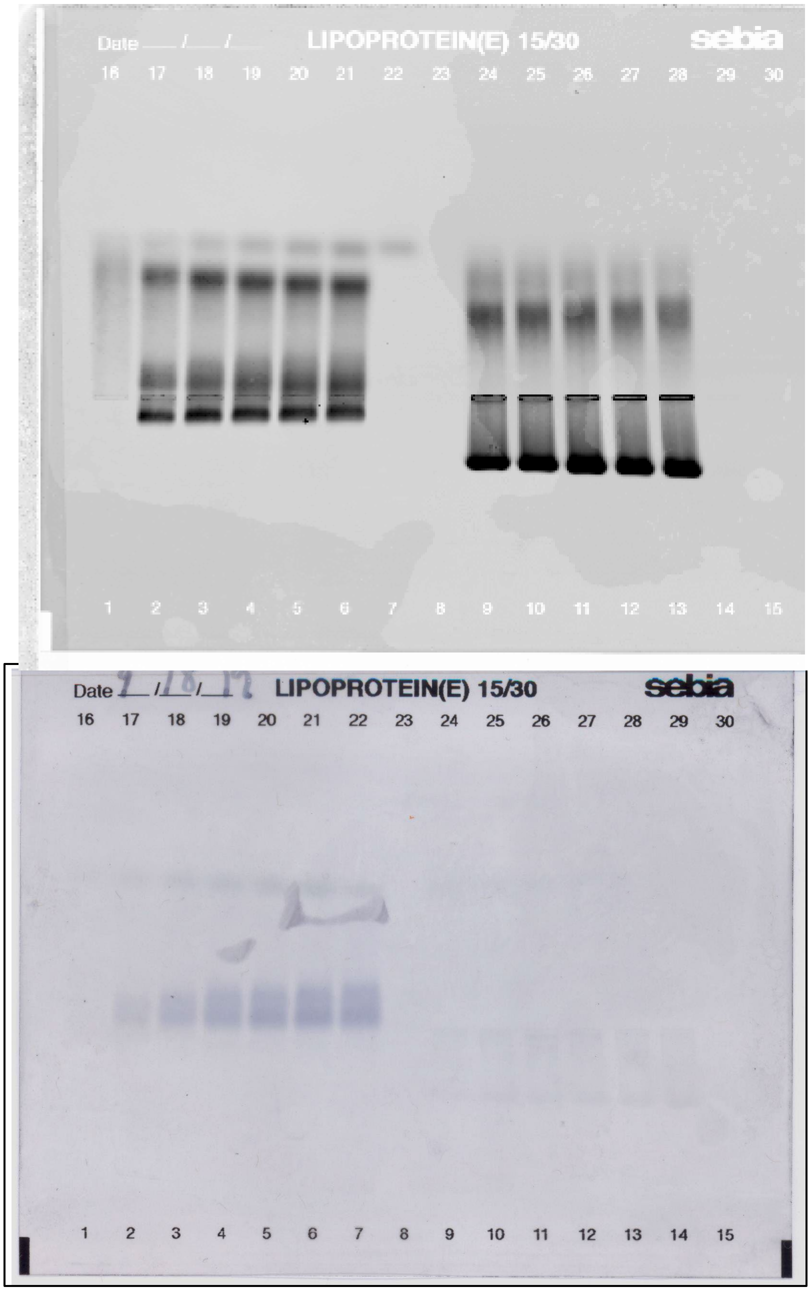

1 2 3 4 5 6 7 8 9 10 11 12 13

Figure S1A (Cont.)

Lane 1: Fatty acid free BSA/BODIPY-cholesterol alone.
Lanes 2-6: Fresh human cholestatic plasma (10, 20, 30 ,40, 50 µL plasma) labeled with 100 µL fatty acid free BSA/BODIPY-cholesterol.
Lane 7: Fresh unlabeled human cholestatic plasma (50 µL).
Lane 8: :Fatty acid free BSA/BODIPY-cholesterol alone.
Lanes 9-13: Fresh synthetic LpX (10, 20, 30, 40, 50 µL LpX) labeled with 100 µL fatty acid free BSA/BODIPY-cholesterol.

Figure S1B

BODIPY-Cholesterol Fluorescence

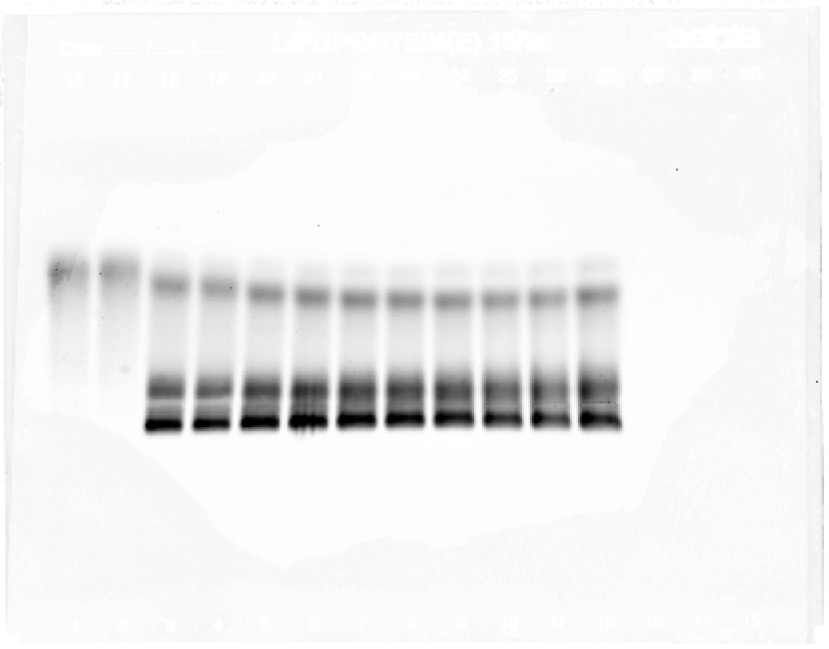

1 2 3 4 5 6 7 8 9 10 11 12

Lanes 1,2: Fatty acid free BSA/BODIPY-cholesterol alone.
Lanes 3,4: Fresh human cholestatic plasma (0.1 µL plasma) labeled with 100 µL fatty acid free BSA/BODIPY-cholesterol.
Lane 5,6: Fresh human cholestatic plasma (1 µL plasma) labeled with 100 µL fatty acid free BSA/BODIPY-cholesterol.
Lane 7,8: Fresh human cholestatic plasma (3 µL plasma) labeled with 100 µL fatty acid free BSA/BODIPY-cholesterol.
Lanes 9,10: Fresh human cholestatic plasma (10 µL plasma) labeled with 100 µL fatty acid free BSA/BODIPY-cholesterol.
Lanes 11,12: Fresh human cholestatic plasma (30 µL plasma) labeled with 100 µL fatty acid free BSA/BODIPY-cholesterol.

Figure S2

BODIPY-Cholesterol Fluorescence

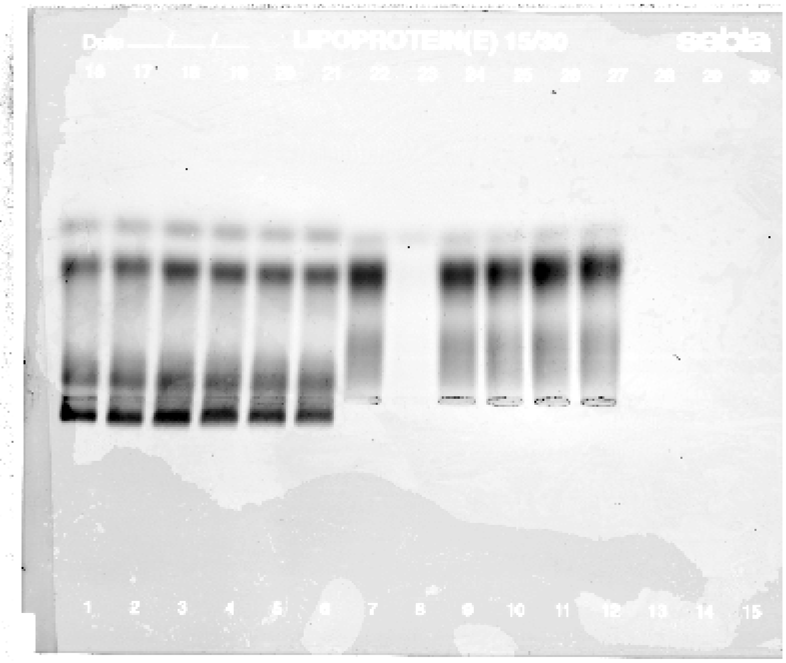

1 2 3 4 5 6 7 8 9 10 11 12

Lanes 1-3: Fresh human cholestatic plasma (25 µL) labeled with 100 µL fatty acid free BSA/BODIPY-cholesterol.
Lanes 4-6: Fresh human cholestatic plasma (25 µL) labeled with 100 µL fatty acid free BSA/BODIPY-cholesterol in the presence of 25 mM trehalose.
Lane 7: Fresh pooled human plasma (25 µL) labeled with 100 µL fatty acid free BSA/BODIPY-cholesterol.
Lane 8: Unlabeled fresh pooled normal human plasma (25 µL).
Lane 9: Fresh pooled normal human plasma (25 µL) labeled with 100 µL fatty acid free BSA/BODIPY-cholesterol.
Lanes 10-12: Fresh pooled normal human plasma (25 µL) labeled with 100 µL fatty acid free BSA/BODIPY-cholesterol in the presence of 25 mM trehalose.

Figure S2 (Cont.)

Sudan Black Staining

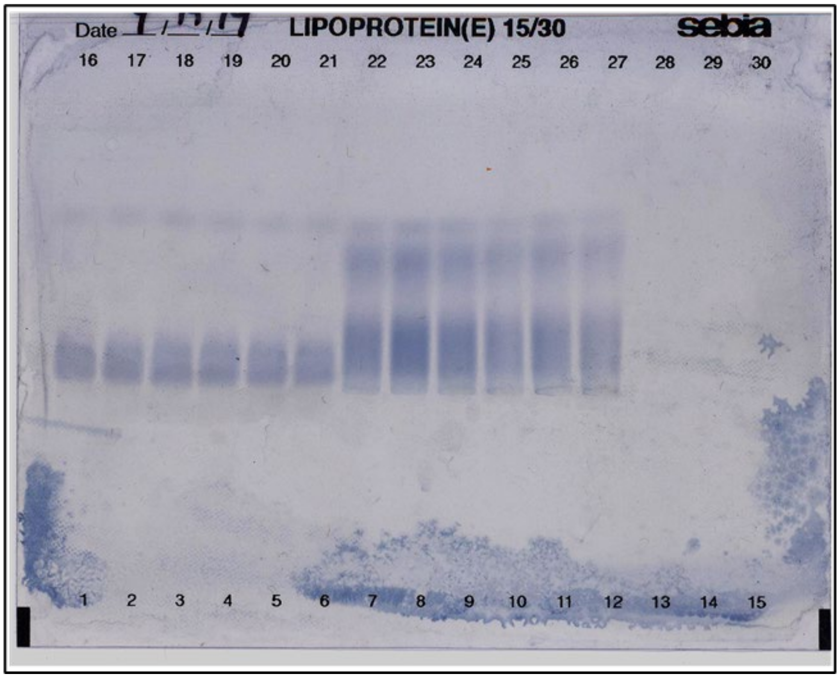

1 2 3 4 5 6 7 8 9 10 11 12

Lanes 1-3: Fresh human cholestatic plasma (25 µL) labeled with 100 µL fatty acid free BSA/BODIPY-cholesterol.
Lanes 4-6: Fresh human cholestatic plasma (25 µL) labeled with 100 µL fatty acid free BSA/BODIPY-cholesterol in the presence of 25 mM trehalose.
Lane 7: Fresh pooled normal human plasma (25 µL) labeled with 100 µL fatty acid free BSA/BODIPY-cholesterol.
Lane 8: Unlabeled fresh pooled normal human plasma (25 µL).
Lane 9: Fresh pooled normal human plasma (25 µL) labeled with 100 µL fatty acid free BSA/BODIPY-cholesterol.
Lanes 10-12: Fresh pooled normal human plasma (25 µL) labeled with 100 µL fatty acid free BSA/BODIPY-cholesterol in the presence of 25 mM trehalose.

Figure S3

Filipin Fluorescence

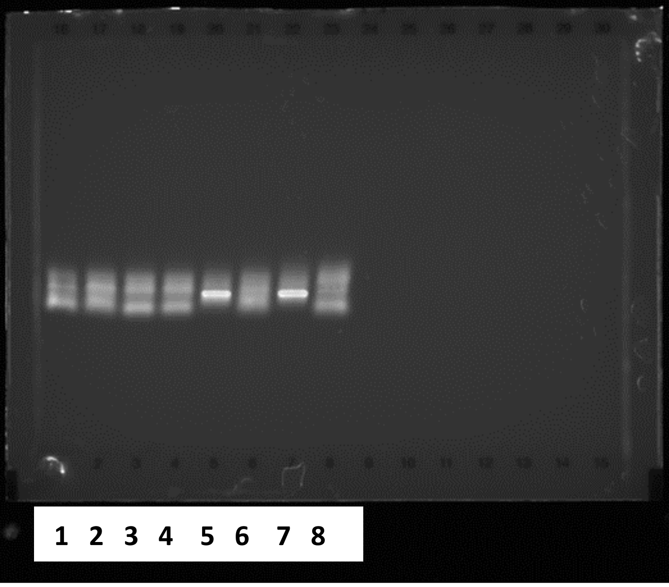

Lanes 1-4: Fresh cholestatic human plasma (25 µL).

Lanes 5-8: Frozen/thawed cholestatic human plasma (25 µL).

Lanes 1,5: Non-treated cholestatic human plasma (25 µL) .

Lanes 2,6: Cholestatic human plasma (25 µL) treated with 25 mM trehalose.

Lanes 3,7: Cholestatic human plasma (25 µL) labeled with 100 µL fatty acid free BSA/BODIPY-cholesterol.

Lanes 4, 8: Cholestatic human plasma (25 µL) labeled with 100 µL fatty acid free BSA/BODIPY-cholesterol treated with 25 mM

Figure S3 (Cont.)

BODIPY-Cholesterol Fluorescence

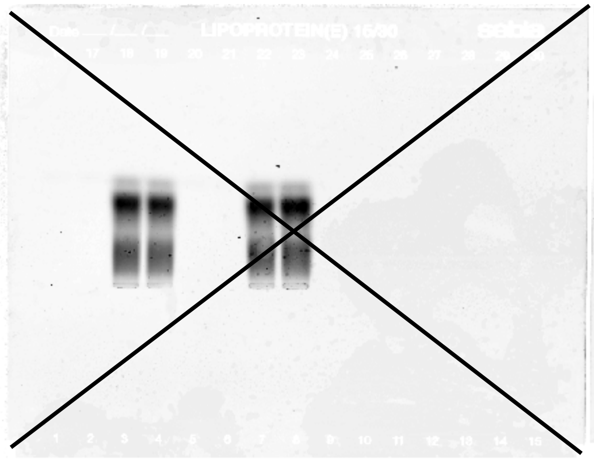

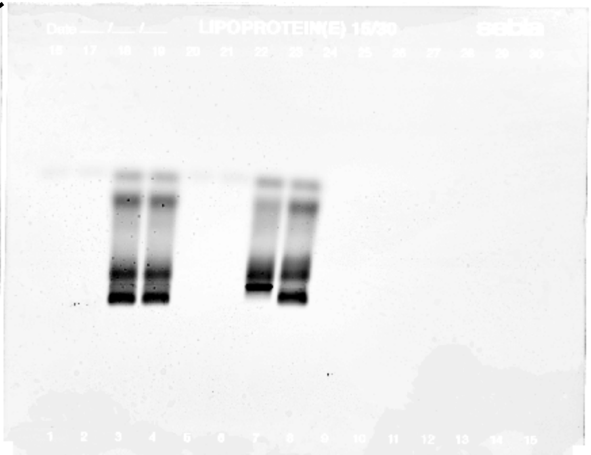

1 2 3 4 5 6 7 8

this gel was not used in manuscript

Lanes 1-4:Fresh cholestatic human plasma (25 µL).

Lanes 5-8: Frozen/thawed cholestatic human plasma (25 µL).

Lanes 1,5: Non-treated cholestatic human plasma (25 µL).

Lanes 2,6: Cholestatic human plasma (25 µL) treated with 25 mM trehalose.

Lanes 3,7: Cholestatic human plasma labeled (25 µL) with 100 µL fatty acid-free BSA/BODIPY-cholesterol.

Lanes 4, 8: : Cholestatic human plasma labeled (25 µL) with 100 µL fatty acid-free

Figure S4

Merged BODIPY-cholesterol (green) and Lissaminerhodamine-phosphatidylethanolamine (red) fluorescence

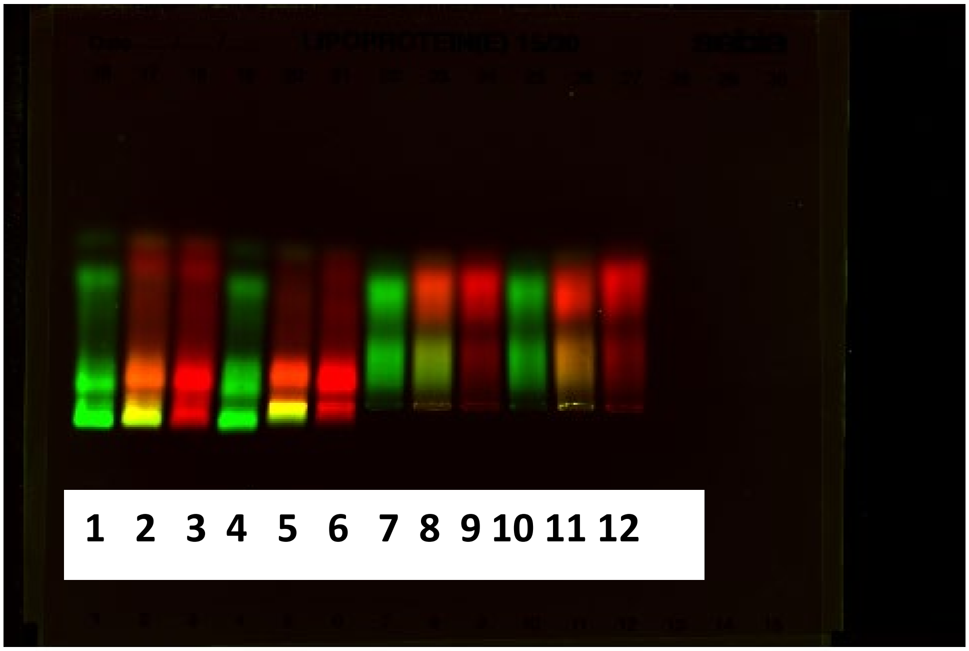

Lanes 1-3: Fresh cholestatic human plasma (25 µL) treated with 25 mM trehalose.

Lanes 4-6: Frozen/thawed cholestatic human plasma (25 µL) treated with 25 mM trehalose.

Lanes 7-9: Fresh pooled normal human plasma (25 µL) treated with 25 mM trehalose.

Lanes 10-12: Frozen/thawed pooled normal human plasma (25 µL) treated with 25 mM trehalose.

Lanes 1,4,7,10: Plasma labeled (25 µL) with 100 µL fatty acid free BSA/BODIPY-cholesterol.

Lanes 3,6,9,12: Plasma labeled (25 µL) with 100 µL fatty acid free BSA/ Lissaminerhodamine-phosphatidylethanolamine.

Lanes 2,5,8,11: Plasma labeled (25 µL) with 100 µL fatty acid free BSA/ BODIPY-cholesterol/Lissaminerhodamine-phosphatidylethanolamine.

Figure S4 (Cont.)

BODIPY-Cholesterol Fluorescence

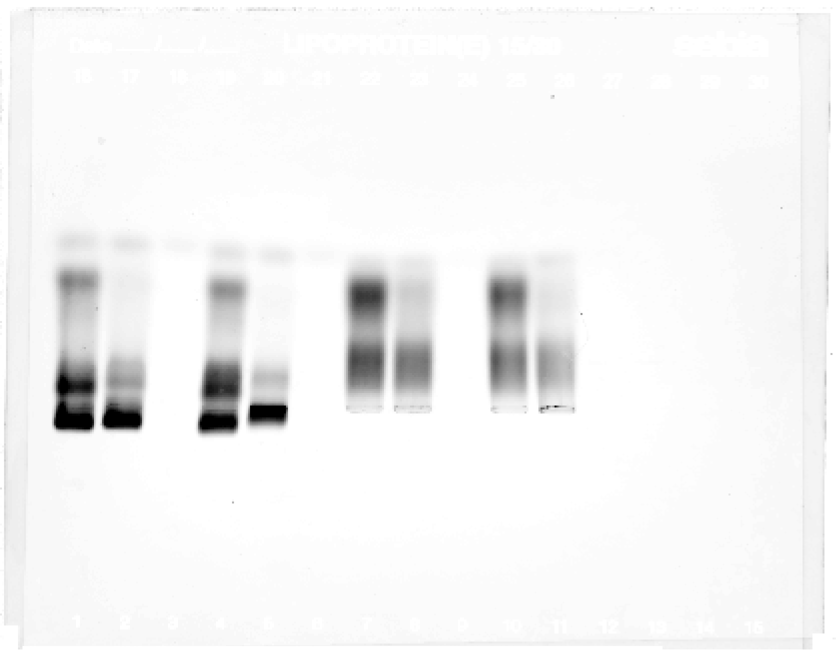

1 2 3 4 5 6 7 8 9 10 11 12

Lanes 1-3: Fresh cholestatic human plasma (25 µL) treated with 25 mM trehalose.

Lanes 4-6: Frozen/thawed cholestatic human plasma (25 µL) treated with 25 mM trehalose.

Lanes 7-9: Fresh pooled normal human plasma (25 µL) treated with 25 mM trehalose.

Lanes 10-12: Frozen/thawed pooled normal human plasma (25 µL) treated with 25 mM trehalose.

Lanes 1,4,7,10: Plasma labeled (25 µL) with 100 µL fatty acid free BSA/BODIPY-cholesterol.

Lanes 3,6,9,12: Plasma labeled (25 µL) with 100 µL fatty acid free BSA/ Lissaminerhodamine-phosphatidylethanolamine.

Lanes 2,5,8,11: Plasma labeled (25 µL) with 100 µL fatty acid free BSA/ BODIPY-cholesterol/Lissaminerhodamine-phosphatidylethanolamine.

Figure S4 (Cont.)

Lissaminerhodamine-phosphatidylethanolamine fluorescence

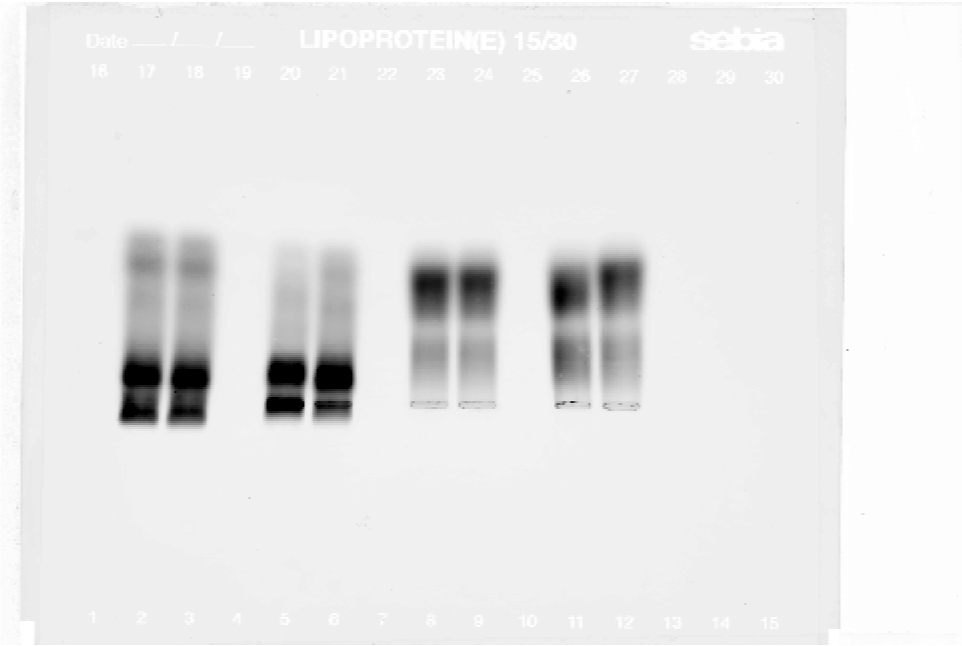

1 2 3 4 5 6 7 8 9 10 11 12

Lanes 1-3: Fresh cholestatic human plasma (25 µL) treated with 25 mM trehalose.

Lanes 4-6: Frozen/thawed cholestatic human plasma (25 µL) treated with 25 mM trehalose.

Lanes 7-9: Fresh pooled normal human plasma (25 µL) treated with 25 mM trehalose.

Lanes 10-12: Frozen/thawed pooled normal human plasma (25 µL) treated with 25 mM trehalose.

Lanes 1,4,7,10: Plasma labeled (25 µL) with 100 µL fatty acid free BSA/BODIPY-cholesterol.

Lanes 3,6,9,12: Plasma labeled (25 µL) with 100 µL fatty acid free BSA/ Lissaminerhodamine-phosphatidylethanolamine.

Lanes 2,5,8,11: Plasma labeled (25 µL) with 100 µL fatty acid free BSA/ BODIPY-cholesterol/Lissaminerhodamine-phosphatidylethanolamine.

Figure S4 (Cont.)

Sudan Black Staining

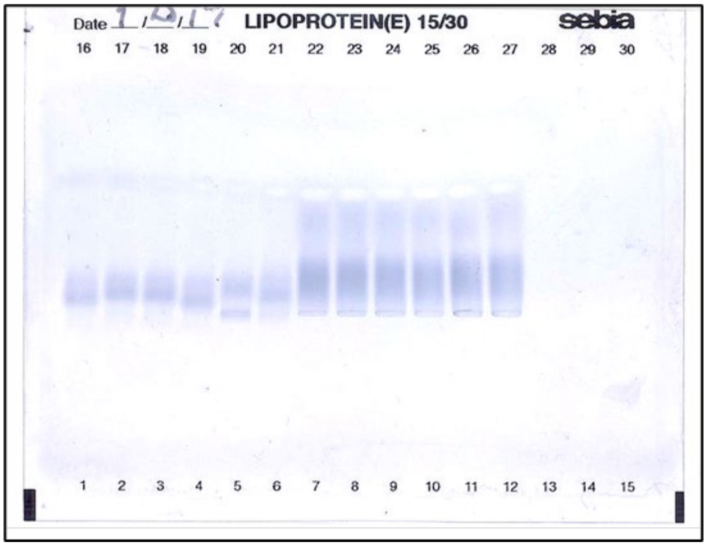

1 2 3 4 5 6 7 8 9 10 11 12

Lanes 1-3: Fresh cholestatic human plasma (25 µL) treated with 25 mM trehalose.

Lanes 4-6: Frozen/thawed cholestatic human plasma (25 µL) treated with 25 mM trehalose.

Lanes 7-9: Fresh pooled normal human plasma (25 µL) treated with 25 mM trehalose.

Lanes 10-12: Frozen/thawed pooled normal human plasma (25 µL) treated with 25 mM trehalose.

Lanes 1,4,7,10: Plasma labeled (25 µL) with 100 µL fatty acid free BSA/BODIPY-cholesterol.

Lanes 3,6,9,12: Plasma labeled (25 µL) with 100 µL fatty acid free BSA/ Lissaminerhodamine-phosphatidylethanolamine.

Lanes 2,5,8,11: Plasma labeled (25 µL) with 100 µL fatty acid free BSA/ BODIPY-cholesterol/Lissaminerhodamine-phosphatidylethanolamine.

Figure S5A

Filipin Fluorescence

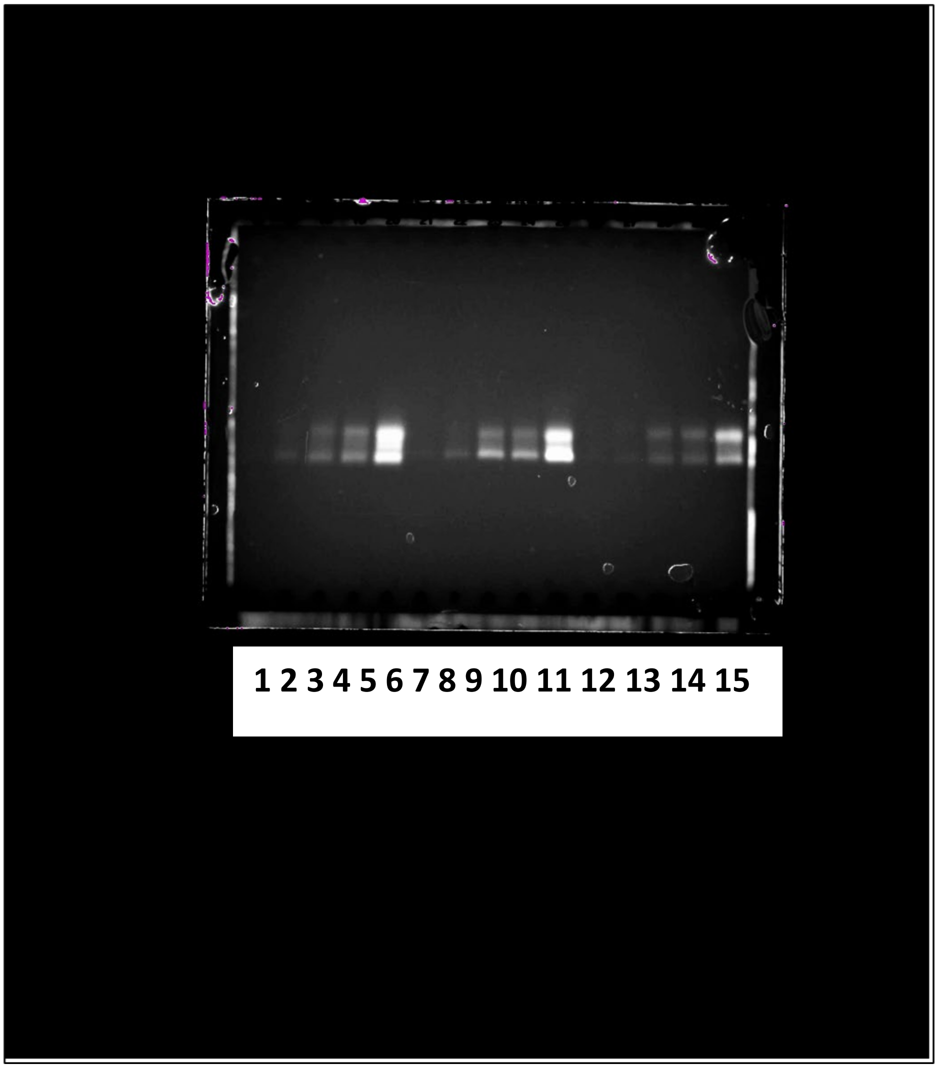

Lanes 1-5: Fresh human FLD plasma from patient #1 (0.1, 0.3, 1,3, or 10 µL) was incubated with BODIPY-cholesterol complexed to fatty acid-free BSA (100 µL) in the presence of 25 mM trehalose.

Lanes 6-10: Fresh human FLD plasma from patient #2 (0.1, 0.3, 1,3, or 10 µL) was incubated with BODIPY-cholesterol complexed to fatty acid-free BSA (100 µL) in the presence of 25 mM trehalose.

Lanes 11-15: Fresh human FLD plasma from patient #3 (0.1, 0.3, 1,3, or 10 µL) was incubated with BODIPY-cholesterol complexed to fatty acid-free BSA (100 µL) in the presence of 25 mM trehalose.

Figure S5A (Cont.)

BODIPY-Cholesterol Fluorescence

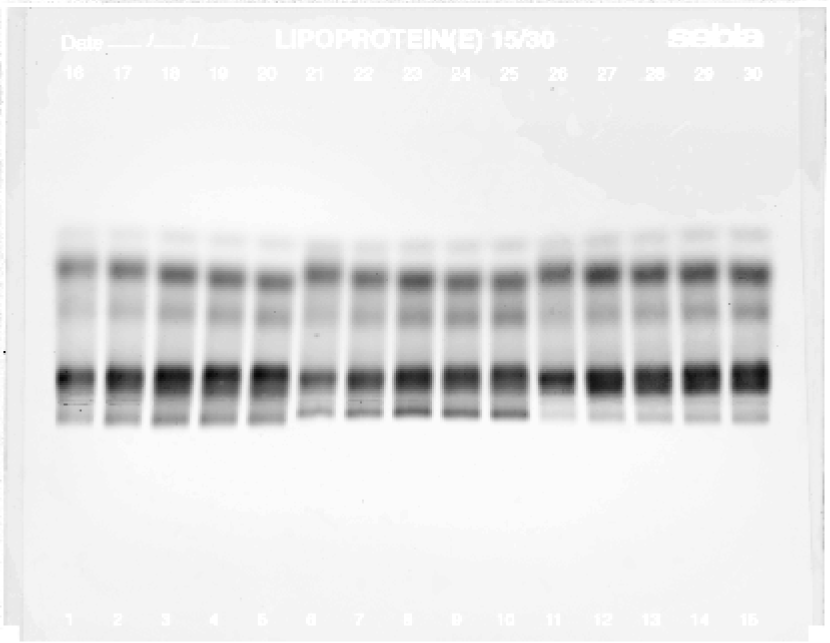

1 2 3 4 5 6 7 8 9 10 11 12 13 14 15

Lanes 1-5: Fresh human FLD plasma from patient #1 (0.1, 0.3, 1,3, or 10 µL) incubated with BODIPY-cholesterol complexed to fatty acid-free BSA (100 µL) in the presence of 25 mM trehalose.

Lanes 6-10: Fresh human FLD plasma from patient #2 (0.1, 0.3, 1,3, or 10 µL) incubated with BODIPY-cholesterol complexed to fatty acid-free BSA (100 µL) in the presence of 25 mM trehalose.

Lanes 11-15: Fresh human FLD plasma from patient #3 (0.1, 0.3, 1,3, or 10 µL) incubated with BODIPY-cholesterol complexed to fatty acid-free BSA (100 µL) in the presence of 25 mM trehalose.

Figure S5B

Filipin Fluorescence

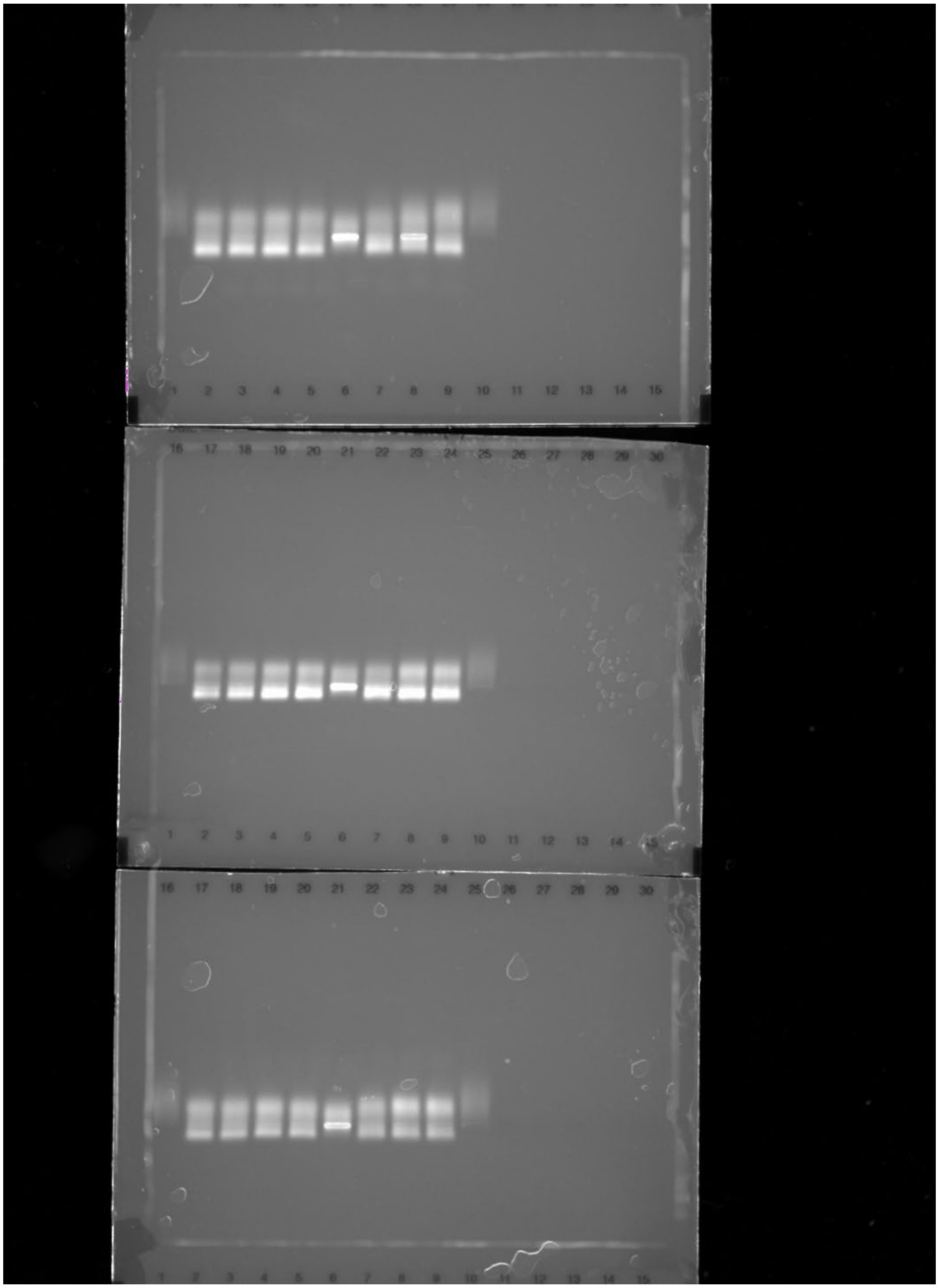

1 2 3 4 5 6 7 8 9 10

Figure S5B (Cont.)

Filipin Fluorescence

TOP GEL:
Lane 1: Fresh normal human plasma (25 µL) incubated with BODIPY-cholesterol complexed to fatty acid-free BSA (100 µL) in the presence of 25 mM trehalose.
Lane 2: Fresh human FLD plasma from patient #1 (25 µL) incubated with saline only.
Lane 3: Fresh human FLD plasma from patient #1 (25 µL) incubated with 25 mM trehalose.
Lane 4: Fresh human FLD plasma from patient #1 (25 µL) incubated with BODIPY-cholesterol complexed to fatty acid-free BSA (100 µL).
Lane 5: Fresh human FLD plasma from patient #1 (25 µL) incubated with BODIPY-cholesterol complexed to fatty acid-free BSA (100 µL) in the presence of 25 mM trehalose.
Lane 6: Frozen/thawed human FLD plasma from patient #1 (25 µL) incubated with saline only.
Lane 7: Frozen/thawed human FLD plasma from patient #1 (25 µL) incubated with 25 mM trehalose.
Lane 8: Frozen/thawed human FLD plasma from patient #1 (25 µL) incubated with BODIPY-cholesterol complexed to fatty acid-free BSA (100 µL).
Lane 9: Frozen/thawed FLD plasma from patient #1 (25 µL) incubated with BODIPY-cholesterol complexed to fatty acid-free BSA (100 µL) in the presence of 25 mM trehalose.
Lane 10: Frozen/thawed normal human plasma (25 µL) incubated with BODIPY-cholesterol complexed to fatty acid-free BSA (100 µL) in the presence of 25 mM trehalose.

MIDDLE GEL:
Lane 1: Fresh normal human plasma (25 µL) incubated with BODIPY-cholesterol complexed to fatty acid-free BSA (100 µL) in the presence of 25 mM trehalose.
Lane 2: Fresh human FLD plasma from patient #2 (25 µL) incubated with saline only.
Lane 3: Fresh human FLD plasma from patient #2 (25 µL) incubated with 25 mM trehalose.
Lane 4: Fresh human FLD plasma from patient #2 (25 µL) incubated with BODIPY-cholesterol complexed to fatty acid-free BSA (100 µL).
Lane 5: Fresh human FLD plasma from patient #2(25 µL) incubated with BODIPY-cholesterol complexed to fatty acid-free BSA (100 µL) in the presence of 25 mM trehalose.
Lane 6: Frozen/thawed human FLD plasma from patient #2 (25 µL) incubated with saline only.
Lane 7: Frozen/thawed human FLD plasma from patient #2 (25 µL) incubated with 25 mM trehalose.
Lane 8: Frozen/thawed human FLD plasma from patient #2 (25 µL) incubated with BODIPY-cholesterol complexed to fatty acid-free BSA (100 µL).
Lane 9: Frozen/thawed FLD plasma from patient #2 (25 µL) incubated with BODIPY-cholesterol complexed to fatty acid-free BSA (100 µL) in the presence of 25 mM trehalose.
Lane 10: Frozen/thawed normal human plasma (25 µL) incubated with BODIPY-cholesterol complexed to fatty acid-free BSA (100 µL) in the presence of 25 mM trehalose.

BOTTOM GEL:
Lane 1: Fresh normal human plasma (25 µL) incubated with BODIPY-cholesterol complexed to fatty acid-free BSA (100 µL) in the presence of 25 mM trehalose.
Lane 2: Fresh human FLD plasma from patient #3 (25 µL) incubated with saline only.
Lane 3: Fresh human FLD plasma from patient #3 (25 µL) incubated with 25 mM trehalose.
Lane 4: Fresh human FLD plasma from patient #3 (25 µL) incubated with BODIPY-cholesterol complexed to fatty acid-free BSA (100 µL).
Lane 5: Fresh human FLD plasma from patient #3 (25 µL) incubated with BODIPY-cholesterol complexed to fatty acid-free BSA (100 µL) in the presence of 25 mM trehalose.
Lane 6: Frozen/thawed human FLD plasma from patient #3 (25 µL) incubated with saline only.
Lane 7: Frozen/thawed human FLD plasma from patient #3 (25 µL) incubated with 25 mM trehalose.
Lane 8: Frozen/thawed human FLD plasma from patient #3 (25 µL) incubated with BODIPY-cholesterol complexed to fatty acid-free BSA (100 µL).
Lane 9: Frozen/thawed FLD plasma from patient #3 (25 µL) incubated with BODIPY-cholesterol complexed to fatty acid-free BSA (100 µL) in the presence of 25 mM trehalose.
Lane 10: Frozen/thawed normal human plasma (25 µL) incubated with BODIPY-cholesterol complexed to fatty acid-free BSA (100 µL) in the presence of 25 mM trehalose.

Figure S5B Cont

BODIPY-Cholesterol Fluorescence

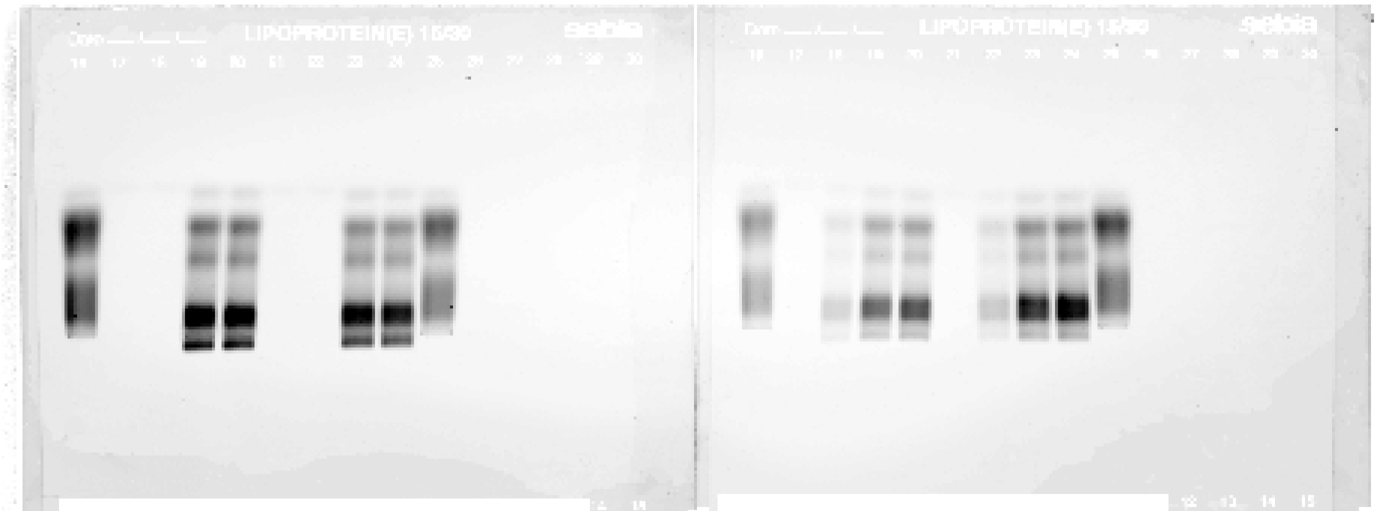

1 2 3 4 5 6 7 8 9 10 11 12 13 14 15 16 17 18 19 20

THE SAME IMAGING PARAMETERS WERE USED FOR ALL BODIPY-CHOLESTEROL FLUORESCENCE SCANS IN FIGURE 5B

Lane 1: Fresh normal human plasma (25 µL) incubated with BODIPY-cholesterol complexed to fatty acid-free BSA (100 µL) in the presence of 25 mM trehalose.
Lane 2: Fresh human FLD plasma from patient #1 (25 µL) incubated with saline only.
Lane 3: Fresh human FLD plasma from patient #1 (25 µL) incubated with 25 mM trehalose.
Lane 4: Fresh human FLD plasma from patient #1 (25 µL) incubated with BODIPY-cholesterol complexed to fatty acid-free BSA (100 µL).
Lane 5: Fresh human FLD plasma from patient #1 (25 µL) incubated with BODIPY-cholesterol complexed to fatty acid-free BSA (100 µL) in the presence of 25 mM trehalose.
Lane 6: Frozen/thawed human FLD plasma from patient #1 (25 µL) incubated with saline only.
Lane 7: Frozen/thawed human FLD plasma from patient #1 (25 µL) incubated with 25 mM trehalose.
Lane 8: Frozen/thawed human FLD plasma from patient #1 (25 µL) incubated with BODIPY-cholesterol complexed to fatty acid-free BSA (100 µL).
Lane 9: Frozen/thawed FLD plasma from patient #1 (25 µL) incubated with BODIPY-cholesterol complexed to fatty acid-free BSA (100 µL) in the presence of 25 mM trehalose.
Lane 10: Frozen/thawed normal human plasma (25 µL) incubated with BODIPY-cholesterol complexed to fatty acid-free BSA (100 µL) in the presence of 25 mM trehalose.

Lane 11: Fresh normal human plasma (25 µL) incubated with BODIPY-cholesterol complexed to fatty acid-free BSA (100 µL) in the presence of 25 mM trehalose.
Lane 12: Fresh human FLD plasma from patient #2 (25 µL) incubated with saline only.
Lane 13: Fresh human FLD plasma from patient #2 (25 µL) incubated with 25 mM trehalose.
Lane 14: Fresh human FLD plasma from patient #2 (25 µL) incubated with BODIPY-cholesterol complexed to fatty acid-free BSA (100 µL).
Lane 15: Fresh human FLD plasma from patient #2 (25 µL) incubated with BODIPY-cholesterol complexed to fatty acid-free BSA (100 µL) in the presence of 25 mM trehalose.
Lane 16: Frozen/thawed human FLD plasma from patient #2 (25 µL) incubated with saline only.
Lane 17: Frozen/thawed human FLD plasma from patient #2 (25 µL) incubated with 25 mM trehalose.
Lane 18: Frozen/thawed human FLD plasma from patient #2 (25 µL) incubated with BODIPY-cholesterol complexed to fatty acid-free BSA (100 µL).
Lane 19: Frozen/thawed FLD plasma from patient #2 (25 µL) incubated with BODIPY-cholesterol complexed to fatty acid-free BSA (100 µL) in the presence of 25 mM trehalose.
Lane 20: Frozen/thawed normal human plasma (25 µL) incubated with BODIPY-cholesterol complexed to fatty acid-free BSA (100 µL) in the presence of 25 mM trehalose.

Figure S5B (Cont.)

BODIPY-Cholesterol Fluorescence

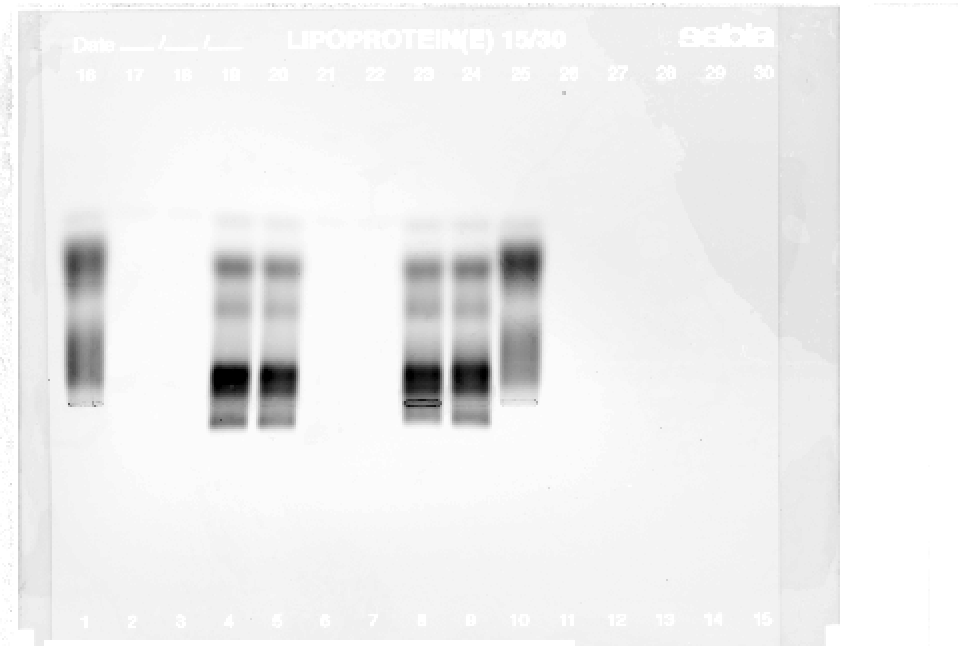

1 2 3 4 5 6 7 8 9 10

Lane 1: Fresh normal human plasma (25 µL) incubated with BODIPY-cholesterol complexed to fatty acid-free BSA (100 µL) in the presence of 25 mM trehalose.
Lane 2: Fresh human FLD plasma from patient #3 (25 µL) incubated with saline only.
Lane 3: Fresh human FLD plasma from patient #3 (25 µL) incubated with 25 mM trehalose.
Lane 4: Fresh human FLD plasma from patient #3 (25 µL) incubated with BODIPY-cholesterol complexed to fatty acid-free BSA (100 µL).
Lane 5: Fresh human FLD plasma from patient #3 (25 µL) incubated with BODIPY-cholesterol complexed to fatty acid-free BSA (100 µL) in the presence of 25 mM trehalose.
Lane 6: Frozen/thawed human FLD plasma from patient #3 (25 µL) incubated with saline only.
Lane 7: Frozen/thawed human FLD plasma from patient #3 (25 µL) incubated with 25 mM trehalose.
Lane 8: Frozen/thawed human FLD plasma from patient #3 (25 µL) incubated with BODIPY-cholesterol complexed to fatty acid-free BSA (100 µL).
Lane 9: Frozen/thawed FLD plasma from patient #3 (25 µL) incubated with BODIPY-cholesterol complexed to fatty acid-free BSA (100 µL) in the presence of 25 mM trehalose.
Lane 10: Frozen/thawed normal human plasma (25 µL) incubated with BODIPY-cholesterol complexed to fatty acid-free BSA (100 µL) in the presence of 25 mM trehalose.
